# Prevalence of placental malaria among asymptomatic pregnant women in Wolkite health center, Gurage zone, Southern Ethiopia

**DOI:** 10.1186/s40794-020-00121-3

**Published:** 2020-10-12

**Authors:** Absra Solomon, Daniel Kahase, Mihret Alemayhu

**Affiliations:** Department of Medical Laboratory Science, College of Medicine and Health Science, Wolkite University, Wolkite, Ethiopia

**Keywords:** Placental malaria, Low birth weight, *Plasmodium falciparum*, *Plasmodium vivax*, Maternal malaria

## Abstract

**Background:**

Placental malaria (PM) is a major public health problem associated with adverse pregnancy outcomes such as low birth weight (LBW), preterm delivery and maternal anemia. The present study is aimed to determine the prevalence of placental malaria among asymptomatic pregnant women in Wolkite health center, Gurage zone, Southern Ethiopia.

**Method:**

Facility-based cross-sectional study was carried out from June 2019 to August 2019. A total of 230 pregnant women were involved in the study where socio-demographic data, medical and obstetric history were collected using pretested structured questionnaires. Blood samples were collected at delivery from maternal capillary, placenta and umbilical cord for the detection of malarial parasite. Maternal hematocrit was determined to screen for anemia.

**Result:**

In this study, the prevalence of placental malaria, peripheral malaria and umbilical cord malaria was 3.9% (9/230), 15.2% (35/230) and 2.6% (6/230) respectively. *Plasmodium falciparum* and *Plasmodium vivax* were detected by microscopy. All babies with positive umbilical cord blood films were born from a mother with placental malaria. Maternal anemia was recorded in 58.3% of the women. In univariate analysis, placental malaria was significantly associated with LBW (*p* < 0.001) unlike parity and maternal anemia.

**Conclusion:**

Placental malaria among asymptomatic pregnant women is low in Wolkite health centre, Gurage zone in Southern Ethiopia. Moreover, placental malaria was strongly associated with LBW. Thus, further strengthening the existing prevention and control activities and screening of asymptomatic pregnant women as part of routine antenatal care service is very essential.

## Background

Malaria in pregnancy (MIP) is a major preventable cause of maternal morbidity and poor birth outcomes in Sub-Saharan Africa [1]. Globally, MIP contributes to about 10,000 maternal deaths and up to 200,000 newborn deaths annually [2]. In 2010, a study in Africa showed that 12.4 million pregnant women were exposed to infection. Of them, 11.4 million women had placental infections without pregnancy-specific protection [3]. World Health Organization (WHO) reported that MIP is responsible for 20% of stillbirths, 11% of newborn deaths and 3.3% of LBW deliveries in sub-Saharan Africa [4].

Placental malaria infection is characterized by the accumulation of parasitized red blood cells (pRBCs) in placental intervillous spaces that express unique membrane-bound proteins from the *P. falciparum* erythrocyte membrane protein-1 (PfEMP-1) to bind to host receptors like chondroitin sulfate A (CSA) in the placental intervillous space leading to placental malaria (PM) [5]. It is associated with increased risk of maternal anemia, LBW, preterm delivery, intrauterine growth restriction, reduced fetal anthropometric parameters, fetal anemia, and congenital malaria [6].

The symptoms and complications of malaria in pregnancy depend on the malaria transmission intensity and the individual’s level of acquired immunity [7]. Pregnant women residing in high malaria transmission areas develop acquired immunity and often have paucisymptomatic or asymptomatic *P. falciparum* infections. However,parasite accumulation in the placenta contributes to maternal anemia and can lead to infiltration of mononuclear cells in the placental intervillous space, which is a risk factor for LBW [8]. On the other hand, pregnant women living in low or unstable malaria transmission areas have relatively little acquired immunity to malaria and are at higher risk of developing severe malaria, which may lead to spontaneous abortion, stillbirth, prematurity, and LBW [8].

As major strategies, WHO has recommended the use of insecticide-treated mosquito nets (ITNs), effective case management of malaria and administration of intermittent preventive treatment with sulfadoxine-pyrimethamine (IPTp-SP) in African countries. Ethiopia does not support IPTp-SP due to cost-effectiveness considerations and low malaria transmission in most parts of the country [9, 10]. The most substantive malaria prevention and control measures in Ethiopia are improving prompt access to diagnostics and treatment, prioritization of ITN use by pregnant women, and enhanced social and behavior change communication activities targeting pregnant women in malaria-endemic areas [10].

Gurage Zone is a malaria-endemic area that has unstable and seasonal malaria transmission. In a previous study, the prevalence of microscopically confirmed malaria cases among symptomatic patients was 8.6% where *P.vivax* accounted for a higher share of 69.7% and *P. falciparum* accounted for 29.3% [11]. In malaria-endemic areas, asymptomatic infection with Plasmodium species is common [12] and such individuals can be the source of malaria transmission to healthy individuals posing serious challenges for malaria control and elimination [13]. We hypothesized that asymptomatic pregnant women are at risk of having undiagnosed placental malaria due to parasite sequestration in the placenta [14]. Hence, the objective of this study was to assess the prevalence of placental malaria among asymptomatic pregnant women in Wolkite health center, Gurage zone, Southern Ethiopia.

## Materials and methods

### Study area

This study was conducted in Wolkite health center, which is found in Wolkite town and located at 158 km southwest of Addis Ababa, the capital city of Ethiopia. Wolkite town is the capital of Gurage Zone, has latitude and longitude of 8°17'N37°47'E and an altitude of 1910- 1935 meters above sea level. Based on the 2007 Ethiopian Census, the town has a total population of 28,856, of whom 15,068 were males and 13,788 were females [15].

Wolkite health center provides service to patients coming from various districts of Gurage zone and an average of 980-1080 women give birth every year.

### Study design and study population

A facility-based cross-sectional study was conducted among asymptomatic pregnant women in Wolkite health center from June 2019 to August 2019. During the study period a total of 230 pregnant women participated in the study. Pregnant women with absence of clinical symptoms mainly fever or history of fever, chills and headache within the last 24 hours, axillary temperatures ≤ 37.5°C and those who were willing to participate and signed the informed consent in the labor ward were included in the study. Participants who took antimalarial drugs, had multiple pregnancies and those with serious delivery complications that led to a referral to other hospitals and those who were unwilling to participate in the study were excluded. Women with *P. falciparum* were treated with Artemether-Lumefantrine (AL) and chloroquine was used for the treatment of *P. vivax.* Infants with umbilical cord malaria were treated with artesunate. All treatments were according to the national treatment guideline. Women who were anemic were treated with ferrous sulfate and folic acid.

### Data collection method

#### Demographic and clinical data collection

After obtaining written informed consent, socio-demographic characteristics of the participants were collected using pre-tested structured questionnaires in the labor ward. An enrollment form containing demographic data, past medical and obstetric history was completed for every participant by trained midwives. A general clinical examination was performed for each woman with reference to antenatal care (ANC) follow up, bed net use, parity, newborn sex. Axillary temperature was measured by an electronic instrument to the nearest 0.1°C at delivery. All newborns were weighted immediately after birth using an electronic scale to the nearest 10 grams and newborn sex was recorded. Newborns were classified as normal birth weight (≥2500 g, regardless of gestational age) or LBW (<2500 g) according to the WHO guidelines [16].

#### Laboratory investigation

##### Maternal peripheral, placental and umbilical cord blood sample collection

At delivery, placental blood films were prepared by the incision of approximately 1.5 cm of the placenta on the maternal side and a drop of blood was placed on a slide. The umbilical cord blood samples were obtained by wiping away excess blood from a clamped cord to avoid contamination with maternal blood. Then, a small cut was made with a lancet and a drop of blood was taken to prepare blood film. Maternal capillary blood samples were collected for blood film and hematocrit determination. All specimens were correctly labeled and sent to the hematology laboratory for hematocrit analysis.

##### Hematocrit determination

Packed cell volume (PCV) of maternal peripheral blood collected after delivery was determined using the standard method. Based on their PCV value women were considered as anemic if PCV is < 33% and were classified as mild (PCV 27–32.9%), moderate (21.0–26.9%) and severe (PCV < 21%) [17].

##### Blood film preparation and examination

Even though placental histology is a gold standard method to diagnose placental malaria [14] due to limited resources and expertise, Giemsa stained blood smear microscopy method was employed.

Both thick and thin smears of the maternal peripheral blood, placental blood and umbilical cord blood were prepared. After proper labeling and air-drying, thin films were fixed with absolute methanol for 5 s while both thin and thick films were stained with freshly prepared 3% Giemsa solution diluted with buffered water (PH 7.1–7.2) for 30 min and examined under 100X magnification [18]. Thick blood films were used to detect the presence of asexual stages of Plasmodium species while thin blood films were used for species identification when thick blood films were positive. Blood films were examined individually by two experienced laboratory technologists. Any discordant results were resolved by a third reader, a senior parasitologist from Wolkite University.

According to WHO, parasite density estimation from parasites counted against high power field (HPF) (100× objective and 10× eyepieces) on the thick film was calculated as [19];

$$ Parasite\ density\  per\ \mu L= Number\ of\ parasites\ counted\div \left( Number\ of\ HPFs\times Volume\ of\ blood\  per\  HPF\right) $$

The standard volume of blood per HPF is assumed to be 0.002. A negative blood film was reported if no parasite were identified after 100 HPFs were observed.

### Data management and quality control

Data quality was ensured using standardized data collection materials, pre-tested questionnaires, properly trained data collectors and intensive supervision during data collection. Pre-analytical, analytical and post-analytical stages of quality assurance that are incorporated in standard operating procedures (SOPs) were strictly followed while performing laboratory analysis.

### Data analysis

The collected data were entered and analyzed using the Statistical Package for Social Sciences (SPSS) version 20 software. Univariate analysis was performed to determine the association of placental malaria with pregnancy and neonatal outcomes. Pearson Chi-square test or Fisher’s exact test was used for categorical variables whereas Student’s t-test was used for continuous variables. A *p*-value <0.05 was considered statistically significant.

## Results

### Characteristics of the study population

Two hundred forty-two women visited Wolkite health center for delivery from June 2019 to August 2019. Of these, 12 were excluded due to prenatal death, twin delivery and referral to a different hospital due to delivery complications. From 230 normal deliveries, 53.9% of the babies were females and 46.1% were males. The age of the participants ranged from 18 to 40 years old with a mean (SD) age of 24.7 (5.1) years. The majority (72.2%) of participants were in the age group of 18–27 years. Sixty-four percent of the participants resided in Wolkite town and more than 90% of the women reported using bed nets during their pregnancy and attended ANC (Table 1).

**Table 1:** Socio-demographic characteristics of the study participant (*n* = 230)

Variables	Frequency (%)
**Age group (years)**	
18–27	166 (72.2)
28–37	60(26.1)
≥ 38	4 (1.7)
**Residence**	
Wolkite	149 (64.8)
Abeshge	46 (20)
Kebena	31 (13.5)
Others	4 (1.7)
**Marital status**	
Married	228 (99.1)
Single	2 (0.9)
**Educational status**	
No formal	38 (16.5)
Primary	134 (58.3)
Secondary	44 (19.1)
Tertiary	14 (6.1)
**ANC Visits**	
Yes	211 (91.7)
No	19 (8.3)
**Bed net use**	
Yes	213 (92.6)
No	17 (7.4)
**Parity**	
Primigravida (Para 0)	40 (17.4)
Primipara (Para 1)	23 (10)
Secundipara (Para 2)	75 (32.6)
Multiparity (Para ≥3)	92 (40)
**Newborn Sex**	
Male	106 (46.1)
Female	124 (53.9)

### Prevalence of malaria at the time of delivery

The prevalence of placental, peripheral and umbilical cord malaria was 3.9% (9/230), 15.2% (35/230) and 2.6% (6/230) respectively. *P. falciparum* and *P. vivax* were both detected in the blood films by microscopy. Mixed infection was not detected in the blood films. *P. falciparum* was detected in 1.7% of placental blood films, 5.2% from peripheral blood films and 1.7% from umbilical cord blood films. *P. vivax* was identified in 2.2% of placental blood films, 10% from peripheral blood films and 0.9% from umbilical cord blood films. The range of parasitemia/μl determined from positive placental, peripheral and cord blood films was 2500–9000/ μl, 2500–13,500/ μl and 2000–7500/ μl (Table 2).

**Table 2:** Distribution of malaria parasitemia at the time of delivery

Variables	Sample source		
	Placental blood film, N (%)	Peripheral blood film, N (%)	Umbilical Cord blood film, N (%)
Positive parasitiaemia	9/230 (3.9)	35/230 (15.2)	6/230 (2.6)
**Plasmodium Species**			
*P.falciparum*	4 (1.7)	12 (5.2)	4 (1.74)
*P.vivax*	5 (2.2)	23 (10)	2 (0.9)
Range of parasitiaemia	2500–9000/ μl	2500–13,500/ μl	2000–7500/ μl
**Parasite density/ μl** ^**a**^			
1–999 parasites/μl	–	–	–
1000–9999/μl	9 (100)	32 (91.4)	6 (100)
> 10,000/μl	–	3 (8.57)	
**Newborn Sex**			
Male	3 (1.3)	15 (6.5)	1 (0.4)
Female	6 (2.6)	20 (8.7)	5 (2.2)

^a^ percentage calculated from the positive malaria parasitemia

### Association of placental malaria with maternal and umbilical cord parasitemia

Of the 9 women with placental malaria, 6 had peripheral parasitemia and all babies with positive umbilical cord blood films were born from a mother with placental malaria. Placental parasitemia was strongly associated with peripheral and umbilical cord blood film parasitemias (*p* < 0.001) (Table 3).

**Table 3:** Association of placental malaria with maternal and umbilical cord parasitaemia

Sample source	Total N (%)	Placental blood film, N (%)		*P*
		Positive	Negative	
**Peripheral blood film**				
Positive	35 (15.2)	6 (17.1)	29 (82.9)	<0.001
Negative	195 (84.8)	3 (1.5)	192 (98.4)	
**Umbilical Cord blood film**				
Positive	6 (2.6)	6 (100)	0 (0)	<0.001
Negative	224 (97.4)	3 (1.3)	221 (98.7)	
Total	230	9 (3.9)	221 (96.1)	

### Association of Placental malaria with pregnancy and neonatal outcomes

The mean birth weight (SD) among women with placental malaria was 2.2 (0.2) as compared to those without malaria 3.4 (0.4) and the difference was statistically significant (*p* = 0.04). Of 230 babies delivered, 7.4% (17) had LBW (< 2.5 kg) and 66.7% (6/9) women with placental malaria had LBW babies. The difference was statistically significant (*p* < 0.001).

The mean hematocrit (HCT) among women was 31.0 (6.2) and anemia (HCT < 33%) was recorded in 57.8% (133) of the women. Of these, mild anemia was seen in 61% (81) of the women, moderate anemia in 31.5% (42) women and severe anemia was seen in 7.5% (10) of the women. From 9 women with placental malaria, 8 (88.9%) of them were anemic and among 221 women without placental malaria, 125 (56.6%) of them were anemic. The difference was not statistically significant (*p* = 0.05) (Table 4).

**Table 4:** Association between malaria at delivery and pregnancy and neonatal outcome

Variables	Total N (%)	Placental malaria N (%)		*P* value	Peripheral blood film N (%)		*P* value
		Positive	Negative		Positive	Negative	
**Parity**							
Primigravidae	40 (17.4)	2 (22.2)	38 (17.2)	0.73	5 (14.3)	35 (18)	0.7
Primipara	23 (10)	1 (11.1)	22 (10)		4 (11.4)	19 (9.7)	
Secondgravida	75 (32.5)	4 (44.4)	71 (32.1)		14 (40)	61 (31.3)	
Multigravida	92 (40)	2 (22.2)	90 (40.7)		12 (34.3)	80 (41)	
**Mean birth weight in kg (SD)**^**a**^	–	2.2 (0.2)	3.4 (0.4)	0.04	3.2 (0.5)	3.4 (0.5)	0.015
**Birth weight (kg)**							
< 2.5	17 (7.4)	6 (66.7)	11 (5)	<0.001	7 (20)	10 (5.1)	0.002
≥ 2.5	213 (92.6)	3 (33.3)	210 (95)		28 (80)	185 (94.9)	
**Maternal HCT (%)**							
≥ 33	97 (42.2)	1 (11.1)	96 (43.4)	0.05	11 (31.4)	86 (44.1)	0.1
< 33	133 (57.8)	8 (88.9)	125 (56.6)		24 (68.6)	109 (55.9)	
Total	230	9 (3.9)	221 (96.1)		35 (15.2)	195 (84.8)	

^a^ Student’s t-test was used for mean birth weight

## Discussion

In this study, the prevalence of placental malaria among asymptomatic pregnant women who delivered at Wolkite health center was 3.9%. This is comparable to the previous finding in unstable malaria transmission areas of Ethiopia which was 2.5% [20] but, lower than other studies reported in Nigeria (65.2%) and Sudan (58.9%) using placental blood microscopy [21, 22]. This prevalence variation might be due to differences in geographic location and malaria transmission intensity. According to the 2015 malaria indicator survey in Ethiopia, 73.9% of pregnant women own an ITN in areas below 2000 m [10]. Malaria endemic areas of Gurage zone had reported the higher utilization of ITN by pregnant women (75%) which might be attributed to low prevalence in the study area [23].

In the present study, the prevalence of maternal peripheral malaria was 15.2% which was comparable to a pooled prevalence among pregnant women in Ethiopia (12.7%) and abroad Guinea (15.8%) [24, 25]. There were 32 discordant results between placental and peripheral malaria: 29 infected women had peripheral parasitemia without placental malaria while 3 had placental malaria without peripheral parasitemia. This was consistent with other studies [26, 27]. Malaria infection may be restricted to periphery due to lack of variant surface antigen expression that can cause placental sequestration [28] or in multigravida, women produce antibodies to VAR2CSA that inhibit the binding of pRBCs to CSA which prevent placental parasitemia [29]. In some cases, women may be infected with variants that adhere to other host receptors, including CD36 and ICAM-1, leading to sequestration in other vascular beds. Thus, these variants may not be detected in placental smears [30]. On the other hand, submicroscopic malaria infections are common during pregnancy and more sensitive approaches like placental histology are needed to detect sub-microscopic infections [31].. A certain level of host immunity may also contribute to microscopically undetectable densities [32].

The prevalence of umbilical cord parasitemia in the present study was 2.6% and all positive babies were born from a mother with both peripheral and placental malaria. There was a strong association of placental malaria with umbilical cord parasitemia (p< 0.001). This prevalence was in agreement with the previous study done in unstable area of Ethiopia (1.6%) and abroad Burkina Faso (1.4%) [20, 33] but lower than studies done by Bassey *et al.* and Fehintola *et al.* which reported malaria prevalences of 50.8% and 40% in Nigeria [21, 34]. Congenital malaria is generally considered infrequent in endemic areas. This might be due to several reasons. The placenta acts as a physical barrier preventing infected erythrocytes from reaching the fetal circulation. In the fetal circulation the presence of fetal hemoglobin (HbF) and low free-oxygen tension cause a poor environment for parasite replication [35]. Further, congenital malaria may be acquired antenatally through placental damage during delivery or premature separation of the placenta resulting in the contamination of fetal blood with infected maternal blood at delivery [36].

The prevalence of LBW in the present study was 7.4% which was comparable with studies in other African countries, including Nigeria 6.7% [21] and Tanzania 6.3% [37]. However, it was lower than other studies in Papua New Guinean 56.4% [38], Sudan 56.4% [22], Malawi 12.2% [39] and Burkino faso 11.6% [40]. Differing results in these studies may be due to transmission intensity, ITN use which was reportedly high in this study and the diagnostic methodology used to diagnose PM.

In our finding, placental malaria was strongly associated with LBW (p<0.001). LBW can be caused by either preterm delivery and/or intrauterine growth restriction. It has been explained in the literature that the sequestration of pRBCs, monocytes in the placenta induce altered cytokine profiles and complement activation that cause placental tissue injury that leads to placental insufficiency, fetal growth restriction which results in LBW [41, 42].

The prevalence of anemia was 58.3% in this study with the majority of women having mild anemia. In the present study, there was no statistically significant association of placental malaria with maternal anemia. The prevalence of anemia in this study was higher than studies conducted by Bassey et al. (31.9%) [21] and Cisse et al. (21.7%) [40]. Even though most of the participants attended ANC where they received oral iron supplementation, the prevalence of anemia was still high. The cause of anemia during pregnancy is multi-factorial which might be nutritional (iron and folic acid deficiency) and/or non-nutritional factors like parasitic infection and HIV infection. Another mechanism is that Plasmodium species affect the iron status through reducing absorption, directly consuming iron for survival or sequestrating iron within the malarial pigment hemozoin [6].The present study has some limitations. Placental histology is the “gold standard” for the diagnosis of placental malaria, as placental histology is considerably more sensitive than microscopy. Further, placental histology can differentiate between acute, chronic, and past malaria infections based on the presence of parasites and/or pigment in the placental intervillous space. Without histology it is not possible to ascertain the duration of placental infections.

## Conclusion

The prevalence of placental malaria among asymptomatic pregnant women was low and all babies with positive umbilical cord blood films were born from a mother with placental malaria. Placental malaria was significantly associated with LBW. Thus, further strengthening the existing prevention and control activities and screening of asymptomatic pregnant women as part of routine ANC is essential.

Moreover, future studies with more sensitive diagnostic techniques including placental histology and PCR should be conducted to determine the burden and effect of submicroscopic infection in pregnant women in high transmission areas.

## Data Availability

The data sets used and/or analyzed during the current study are available from the corresponding author on reasonable request.

## Abbreviations

LBW: Low birth weight
LLIN: Long-lasting insecticidal nets
MIP: Malaria in pregnancy
PCV: Packed cell volume
PM: Placental malaria
PRBCs: Parasitized red blood cells
SOPs: Standard Operating Procedures
WHO: World Health Organization
IPTp-SP: Intermittent preventive treatment of malaria in pregnancy with sulfadoxine-pyrimethamine
HPFs: High power fields
ANC: Antenatal care
CSA: Chondroitin sulphate A
HbF: Fetal hemoglobin
